# Research productivity of doctor of physical therapy faculty promoted in the southeastern United States

**DOI:** 10.1080/10872981.2017.1368849

**Published:** 2017-08-23

**Authors:** Marissa A. Littman, James W. Sonne, Gerald V. Smith

**Affiliations:** ^a^ Physical Therapy Program, Department of Health Professions, University of Central Florida, Orlando, FL, USA

**Keywords:** Tenure, promotion, faculty development, physical therapy, DPT

## Abstract

**Background:** Little information exists on the research productivity of successfully promoted tenure-track Doctor of Physical Therapy (DPT) faculty.

**Objective:** To determine the research productivity that typically results in successful promotion.

**Design:** We collected publicly available curriculum vitae (CVs) from faculty currently in accredited DPT programs and who had been successfully promoted from an institution in the southeastern USA from 2000 through 2016.

Total publication count, journal impact factor, funding, citations, and other metrics were analysed from 45 subjects of 22 of the 64 CAPTE-accredited DPT programs in the southeast.

**Results:** None of the studied metrics were normally distributed with time to promotion as determined by a Shapiro-Wilk test. These faculty exhibited a median publication count of 4, range 0 to 43; median of average citation count of 12.4, range 0 to 87.25; median of average journal impact factor of 2.866, range 0 to 6.280; median external funding received of $9910, range $0.00 to $19 543 198; and median author h-index of 3, range 0 to 17. The median number of years before promotion was 6, ranging from 3 to 13 years. Linear regression analysis indicates a poor fit with no significant correlation between years before promotion and any of the studied metrics. No correlation between journal impact factor and number of citations was observed (m = −0.22, *p =* 0.728, R^2^ = 0.0003). Prior to promotion 31% (14 of 45) did not receive external funding and 24% (11 of 45) had a 0 h-index. The Carnegie Classification of the institution did not significantly correlate with research productivity metrics in this dataset (*p =* 0.213).

**Conclusion:** While faculty unsuccessful in promotion were not identifiable using this method, this research can be used by faculty and committees to evaluate research productivity against regional data and promote competitive standards with peer institutions.

**Abbreviations:** CAPTE: Commission on Accreditation in Physical Therapist Education; DPT: Doctor of Physical Therapy

## Introduction

Currently little to no research exists on the productivity of physical therapist educators who have attained promotion in CAPTE (Commission on Accreditation in Physical Therapist Education) accredited DPT programs and departments. Here we investigated research productivity of tenure-track faculty in CAPTE accredited DPT programs who have been promoted from Assistant Professor to Associate Professor during the year 2000 through 2016 in the southeastern region of the USA.

The year 2000 is important in the history of physical therapy education as the House of Delegates of the American Physical Therapy Association (APTA) created a new vision for the physical therapy profession in which the entry-level degree would become the Doctorate of Physical Therapy. Prior to that, the bachelor’s level, or later the master’s level, degree was considered entry-level. In 2000, APTA stated that ‘physical therapy, by 2020, will be provided by physical therapists who are doctors of physical therapy’ [1,2] causing significant changes in the depth and breadth of the entry-level education and the accrediting processes, resulting in a higher level of faculty credentialing along with institutional expectations of faculty research productivity.

Research productivity is frequently determined by analyzing the number of peer-reviewed published articles, the publishing journal’s impact factor and citation count of these published works, author h-index [3], and the research funding received by junior professors up to the time of being promoted to Associate Professor. While there is much discussion as to the nature of these measures as valid means of determining productivity, this article only attempts to measure these often used metrics to compare the results against recognized standards in other professions and previous publications of research productivity [4,5]. However, it should be noted that citation rates and article visibility, along with the number of co-authors and the curation of journal publications can vary greatly between fields, disciplines, and sub-disciplines [4]. As physical therapy programs and departments incorporate a broad, interdisciplinary approach – which is reflected in their faculty’s degrees, credentials, age, experience in academia, and resource availability – these issues may be valid points of concern especially when considering promotion. As of the 2015–2016 academic year, there are a reported 2437 full-time and 282 part-time core faculty within CAPTE accredited DPT programs nationwide [6]. Of these core faculty, 41.9% (1196 faculty) are certified clinical specialists and only 33.2% (809 faculty) have grant funding of any amount. These faculty hold a combined 2845 degrees, 13.9% of which are professional doctoral degrees (i.e. EdD, DSc, etc.), and 45.9% of which are a PhD. Furthermore, 48.1% of DPT program faculty are at the assistant-level and only 25.5% of core DPT faculty have attained tenure. Contrasting DPT programs with MD programs accredited by the AAMC’s LMCE, 72.9% (121 472) faculty hold an MD and 28.5% (47 518) hold a PhD of the 166 713 total MD program faculty, which is comprised of 3.7% (6116) assistant-level faculty with a PhD in a science field, and 41.9% (69 822) assistant-level faculty with an MD [7]. Because the proportion of non-clinical degrees spanning many different disciplines is higher in DPT programs, it is our hope that the broad range of faculty productivity that we present in this article will be used when creating standard criteria for promotion and tenure, and that this data and these issues will be used by applicants and members of tenure committees when reviewing the productivity of physical therapy educators and programs or departments as a whole.

Educational faculty usually undergo a combined evaluation process for both promotion and tenure; however, these are two separate processes. Tenure is an achieved personnel status which is designed to allow faculty members a sense of job security, influence, and academic freedom to conduct scholarship which may not be in alignment with those of their employing institution. Promotion in this context typically describes an elevation in faculty rank from assistant professor to associate professor, or from associate professor to full professor. The process of promotion from assistant professor to associate professor is most often associated with achieving tenure, although it does not always have to be [8]. Faculty members who wish to apply for tenure normally are employed as ‘tenure-track’ faculty and after a substantial amount of time in this position they are subjected to an evaluation process in which their body of work – including publications, presentations, courses taught, and funded research proposals – is evaluated by an institutional committee [9]. This committee then decides whether or not the faculty member should be promoted and/or granted tenure. The time leading up to this evaluation period can be very stressful as the applicant must be sure their body of work and the contents of their curriculum vitae are at the necessary level for advancement. If the applying faculty member is not granted tenure, he or she often loses their faculty position [8,9].

The method of using publicly available curriculum vitae for data collection is not novel; a study assessing increased publication pressure to achieve tenure on new doctoral graduates of educational psychology programs at research universities used the following methodology: ‘a sample of 197 curriculum vitae was retrieved from educational psychology departmental websites across the 24 universities; vitae were coded for year the faculty member completed his/her doctorate, and total number of publications, number of peer-reviewed publications, and number of first-authored, peer-reviewed publications;’ a method and type of data collection similar to those of this study [10]. The decision to collect research productivity data from promoted and tenured DPT professors in specific regions of the country and calculating individual regional findings is supported by previous reporting in the literature of region-specific scholarly productivity [11], and by the notion that ‘the geographical location of the university, too, could affect the resources, barriers, and productivity (e.g., pilot research studies) a faculty member experiences’ [9]. Such factors may include collaborative potential, the types of regional industries local to the institution, physical distance to other research institutions, and economic factors such as institutional funding and available real property assets at the institution. This study follows the regional breakdown of the USA as defined by the Bureau of Economic Analysis, which identifies differences in fixed assets, consumer durables, personal incomes, gross domestic product and other economic metrics among these regions.

The decisions surrounding tenure have a great impact on the life and career of the applying faculty member, the program and department, universities and the profession; as such, a guideline to aid the committee members who make these decisions would be very beneficial to the candidate, the committee members, and the field of physical therapy as a whole. Furthermore, this study would also allow the applying professor to ensure that his or her body of work is of a content likely to lead to promotion and/or tenure before putting their faculty position at risk.

## Methods

Data were collected for this study from institutionally-linked, publicly available curriculum vitae (CV) of tenure-track (non-clinical) DPT professors currently employed at a CAPTE accredited DPT program and who earned promotion from the years 2000 through 2016 at a university located in the southeastern (SE) USA. The southeastern region was considered to include the states Alabama (AL), Arkansas (AR), Florida (FL), Georgia (GA), Kentucky (KY), Louisiana (LA), Mississippi (MS), North Carolina (NC), South Carolina (SC), Tennessee (TN), Virginia (VA), and West Virginia (WV). Subjects who did not attain tenure simultaneously with promotion were included in the study data. A total of 45 subjects with publicly available CVs who received promotion at 22 of the institutions in these states met these inclusion criteria and were used in this study.

Research productivity factors used in this study include number of publications, citation counts (including self-citation), journal impact factor, h-index at time of promotion, funding received, and the number of years until promotion since first attaining Assistant Professor rank. Only peer-reviewed manuscripts indexed by Thomson Reuters Web of Science and published during the years the subject served as Assistant Professor were used to determine total publications, number of times those publications were cited, and author h-index based on these publications. The most recent five-year journal impact factor for each subject’s publications was collected from Thomson Reuters Journal Citation Reports (JCR). Awarded funding amount up to and including the year of promotion was collected from the subject’s CV and was included if the subject served as the principal or co-investigator for the funded project.

Each subject’s number of publications, total citations, and journal impact factor were averaged, then the median, mean, and standard deviation (SD) of all subjects’ averages were calculated. The median, mean, and standard deviation of all subjects’ internal and external funding, years until promotion, and h-index at the time of promotion were also calculated. Linear regressions and two-way ANOVA analyses (α = 0.05, β = 0.80) were performed using IBM SPSS Statistics release 24.0.0.0 (32-bit edition). No data points were removed from analysis. Confidence intervals are 95% of the mean where shown. Where observed, overlapping data points were offset horizontally against the ordinal x-axis. Data is presented as ‘MEAN (SD).’

## Results

Forty-five subjects from 22 of 64 CAPTE accredited DPT programs in the southeastern (SE) USA were found to meet all inclusion criteria. The number of universities represented in this study compared with the total CAPTE accredited DPT universities per state are as follows: AL: 2/4, AR: 2/4, FL: 4/12, GA: 3/7, KY: 1/3, LA: 1/2, MS: 0/2, NC: 2/10, SC: 2/2, TN: 2/6, VA: 1/9, WV: 2/3. Of the 45 total subjects in this study, the number of faculty per state and their proportionality in this study is as follows: AL: 4 (8.9%), AR: 5 (11.1%), FL: 5 (11.1%), GA: 4 (8.9%), KY: 2 (4.4%), LA: 2 (4.4%), MS: 0 (0%), NC: 5 (11.1%), SC: 8 (17.8%), TN: 2 (4.4%), VA: 2 (4.4%), WV: 5 (11.1%). The institutions were distributed among Carnegie Classifications M2: 9/45, M1: 5/45, R3: 1/45, R2: 6/45, R1: 14/45, Four-year medical: 10/45. Two subjects (4.4%) did not receive tenure with promotion but are included in the study. None of the 45 subjects had missing data, and all were included in all analyses and calculations. The median years to promotion since first hired as a tenure-track assistant professor was 6 with an average of 7.1 (4.0 SD) and ranging from 3 to 13.

As described in Table 1, the median publication count for DPT faculty promoted from assistant to associate professor in the SE region was 4 with an average of 8.4 (10.9) with a range of 0 to 43 publications. A total of 8 subjects (17.8%) had no indexed publications. The median of total citations was 53 with an average of 146.1 (221.6) ranging from 0 to 935 citations. The most cited single article by a subject in this study received 205 citations. A total of 10 subjects (22.2%) had received zero citations in an indexed publication. The median of the average five-year journal impact factor in which a subject published was 2.866 with an average of 2.513 (1.556) and a range of 0 to 18.021. The median of average citations per manuscript was 12.4 with an average of 13.8 (15.5) and a range from 0 to 40.5. The median total funding received was $54 000.00 with an average of $1 093 941.14 ($3 033 350.26) and a range of $0.00 to $19 543 198.00. The median of external funding was $9910.00 with an average of $1 013 096.40 ($3 031 176.14) ranging from $0.00 to $19 543 198.00. Subjects had a median h-index factor at time of promotion of 3 with an average of 4.2 (4.1) ranging from 0 to 17. A total of 16 of 45 (35.6%) received no external funding and six faculty (13.3%) received up to $8000 external funding prior to promotion, and 11 of 45 (24%) had an h-index of 0 prior to promotion, 8 of which (17.8%) had no indexed publications. A Shapiro-Wilk test indicated that all of the individual faculty data was significantly non-normal in its distribution (*p *< 0.005), supporting the use of median, not mean and standard deviation, for analysis.

**Table 1.** Forty-five subjects who successfully earned promotion from assistant to associate professor were used in this study. Eight subjects (17.8%) had 0 publications and 10 subjects (22.2%) had 0 citations in publications indexed by Thomson Reuters Web of Science while 14 subjects (31%) had received $0.00 in external funding. ‘R2’ column represents linear fit against ‘Years until promotion,’ and the ‘p-value’ column indicates probability of non-correlation with ‘Years until promotion’ using a two-way ANOVA. Overall probability for all metrics in relation to ‘Years until promotion’ was not significant at *p =* 0.426 (F = 1.021). A Shapiro-Wilk test revealed all factors are significantly non-normal in their distribution (p < 0.005), supporting the use of median, not mean.

Research productivity factors	Median	Range	Mean	Std. Dev.	R^2^	*p*-value
Years until promotion	6	3–13	7.1	4.0	n/a	n/a
Publication count	4	0–43	8.4	10.9	0.050	0.141
Total citation count	53	0–935	146.1	221.6	0.041	0.184
Average citation count	12.4	0–40.5	13.8	15.5	0.010	0.520
Author h-index	3	0–17	4.2	4.1	0.036	0.210
Average journal impact factor	2.866	0.000–6.280	2.513	1.556	0.083	0.056
External funding awarded	$9910.00	$0.00 – $19 543 198.00	$1 013 096.40	$3 031 176.14	0.002	0.753

**Figure 1.**
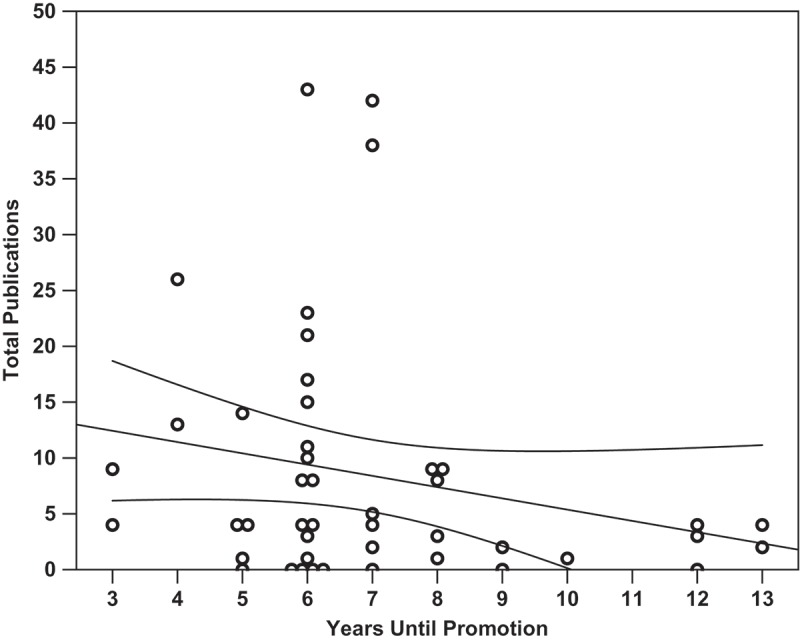
The number of manuscripts an assistant professor published in an indexed source did not significantly correlate with the number of years spent at the rank of assistant professor before promotion (*p =* 0.141, m = −1.01, R^2^ = 0.050). Overlapping data points are offset horizontally against an ordinal x-axis.

**Figure 2.**
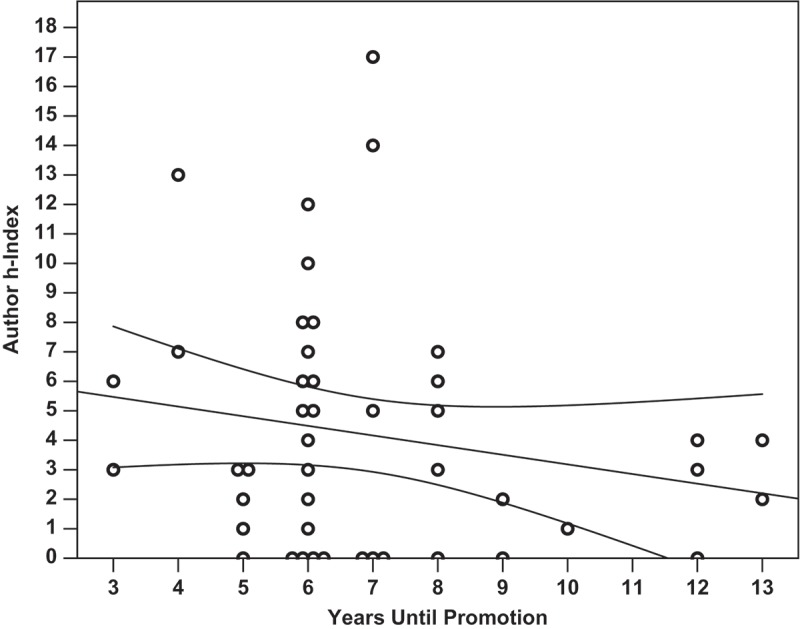
The h-index of an assistant professor at time of promotion did not significantly correlate with the number of years spent prior to promotion to associate. (*p =* 0.210, m = −0.33, R^2^ = 0.036). Overlapping data points are offset horizontally against an ordinal x-axis.

**Figure 3.**
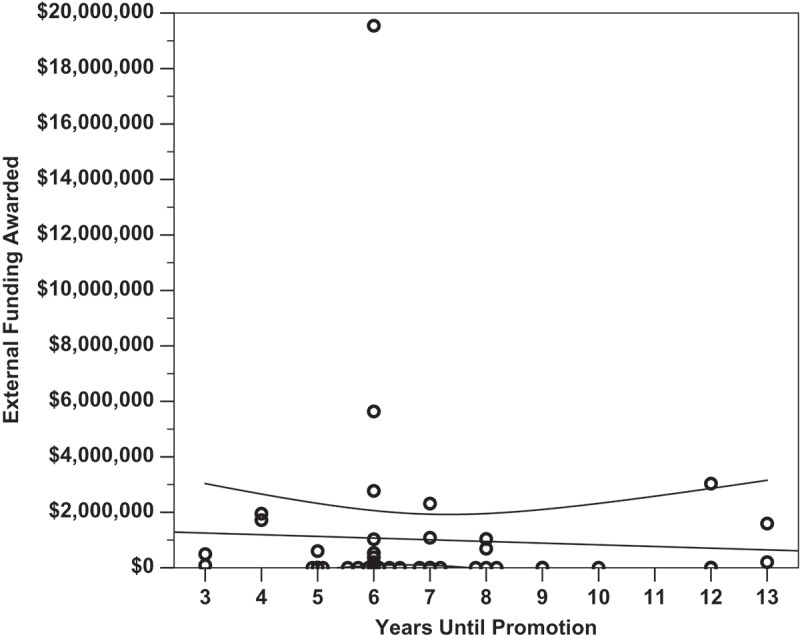
The amount of external funding a faculty member received did not significantly correlate with the number of years they spent earning promotion (*p =* 0.753, m = -$60 700/year, y-int = $1 430 000, R^2^ = 0.002). The data indicates the median amount of external funding a faculty member has received prior to promotion to associate professor is $9910, regardless of the number of years spent earning promotion. A total of 14 of 45 subjects (31%) received $0 in external funding and four subjects (8.9%) received between $1 and $8000. Overlapping data points are offset horizontally against an ordinal x-axis.

**Figure 4.**
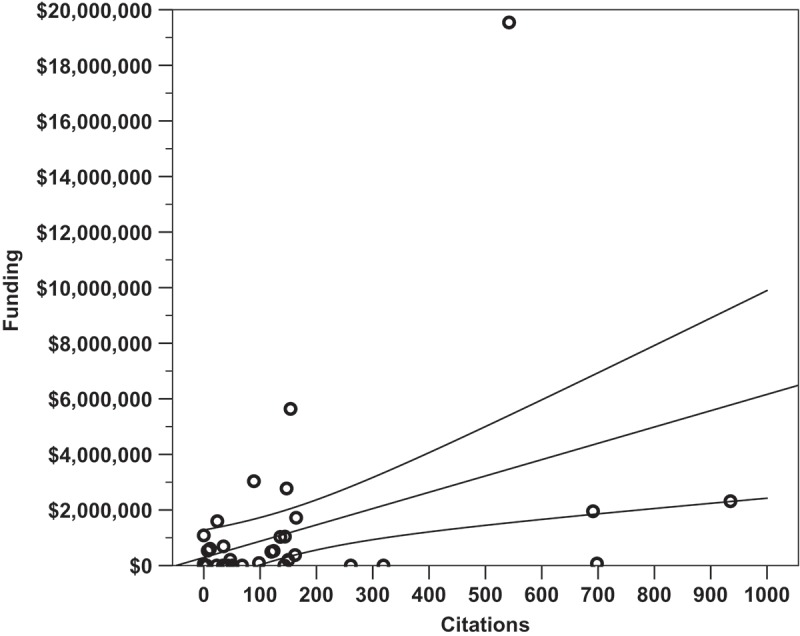
The total number of citations an assistant professor receives is loosely but significantly correlated with the amount of external funding received prior to promotion to associate. (*p =* 0.007, m = $5870 per citation, y-intercept of $288 000, R^2^ = 0.159, CI: ±$910 666.13).

**Figure 5.**
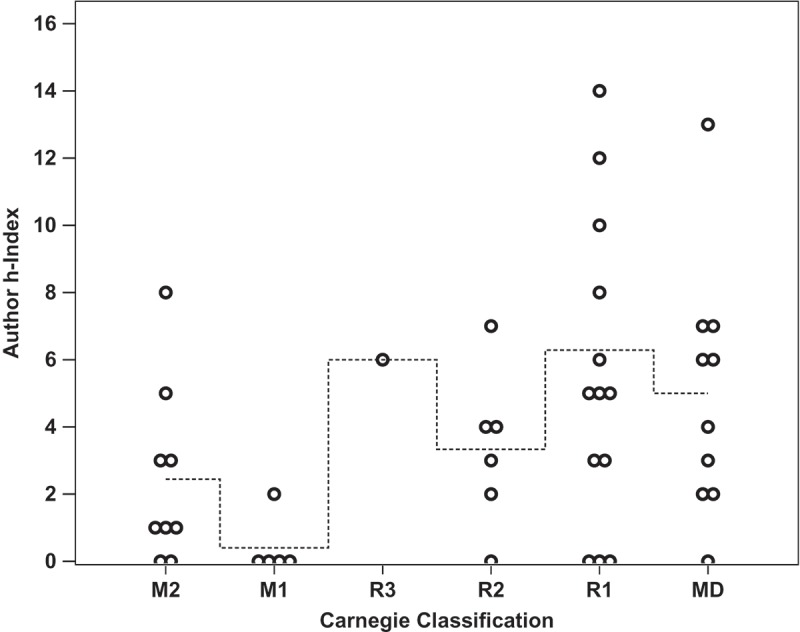
The Carnegie Classification of the promoting institution neither significantly nor closely correlated with the faculty research productivity metrics used in this study, as analysed by linear regression (*p =* 0.213, R^2^ = 0.216). Shown here is the author h-index of faculty within the Carnegie Classifications of their promoting institutions (*p =* 0.040, R^2^ = 0.141; m = 0.83, y-intercept = 1.77). Dashed line represents the stepped mean of faculty h-index faculty from within each classification.

Linear regression analysis was performed to determine which factors most strongly correlated with successful promotion to associate professor. Overall, none of the identified metrics closely nor significantly correlated with the number of years it took a candidate to receive promotion (*p *= 0.426, R^2^ = 0.139). It was found that the number of publications did not significantly correlate with the number of years spent at the rank of assistant professor (Figure 1, *p *= 0.141, m = −1.01, R^2^ = 0.050). Total citations a faculty member received did not correlate with years until promotion (*p *= 0.184, m = −17.2, R^2^ = 0.041), and the h-index of a faculty member at time of promotion did not correlate with years until promotion (Figure 2, *p *= 0.210, m = −0.33, R^2^ = 0.036). The amount of external funding a faculty member was awarded did not significantly correlate with the number of years they spent earning promotion (Figure 3, *p *= 0.753, m = −$60,700/year, R^2^ = 0.002). It was found that amount of external funding received did significantly correlate with the number of total citations a researcher received (Figure 4, *p *= 0.007, m = + 2.71 citations per $100 000, y-intercept = 96.13 citations, R^2^ = 0.159). Finally, the Carnegie Classification of the promoting institution did not significantly correlate with the faculty research productivity metrics within this dataset (*p *= 0.213, R^2^ = 0.216). Within this analysis, only author h-index at time of promotion loosely but significantly co-varied with Carnegie Classification (Figure 5, *p =* 0.040, R^2^ = 0.141, m = 0.83, y-intercept = 1.77).

Six of the subjects (13.3%) were also promoted to full professor within the timeframe of the study with a median time to full professor for this subgroup of 6.5 with an average of 6 (3.3 SD). These subjects as a whole exhibited higher funding during their associate professor years than observed for assistant professors. Median external funding prior to promotion to full professor was $937 468.50, mean of $1 891 682.23 ($2 194 542.21) and with a range from $0 to $4 105 500. All other metrics were lower, however; including median total publications of 0.5, mean of 1.3 (1.9), median total citations of 8 with an average of 21.8 (33.7), median h-index during associate professor years of 1, mean of 1.3 (1.3), median of average citations per article of 8, mean 8.4 (9.8), and median journal impact factor of 1.349, mean of 1.559 (1.833).

**Figure 6.**
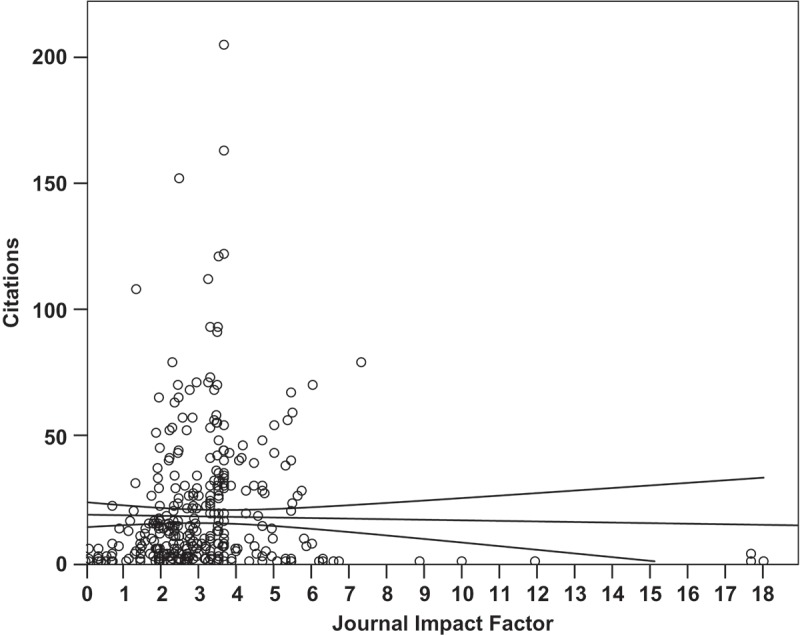
Within this data set, the five-year impact factor of the journal in which a manuscript was published did not significantly correlate with the number of citations that article received (n = 383 manuscripts, *p =* 0.728, m = −0.22, R^2^ = 0.0003).

Using the data from these subjects, we found that the impact factor of the journal in which a manuscript was published did not significantly correlate with the number of citations that article received (Figure 6, *p *= 0.728, m = −0.22, R^2^ = 0.0003), while the number of years since an article was published correlated loosely but significantly (*p *= 0.00000002, m = 1.77 citations per year, y-int = 0 citations, R^2^ = 0.079).

## Discussion

From these forty-five subjects who received promotion and/or tenure at southeastern institutions and are employed by CAPTE accredited DPT programs, we observe that a broad range of scholarship and productivity are likely being considered by different institutions, not sheer cut-offs in the studied metrics. The shortest time to promotion from assistant to associate professor among this group was three years, and the longest was 13 years, while the median was six years. Among these faculty none of the studied metrics identified these individuals as significantly different from the rest of the sample. We found that eight subjects (17.8%) had no indexed publications, consistent with findings in 2009 from self-reported data of entry-level DPT faculty [12]. In contrast, a study of SE program faculty performed in 1987 identified 30% of 127 respondent self-reporting physical therapy faculty as not having authored a scholarly work [11] indicating a possible increase in research productivity over the past 30 years. The faculty that represented the median received 53 total citations for scholarly work in indexed publications; a total of 11 subjects (24.4%) had received zero citations. In terms of external funding, the median from this group of subjects was $9910 prior to promotion. Comprising the two lower quartiles of successfully promoted faculty were 16 subjects (35.6%) that had received no external funding and six subjects (13.3%) that had received external funding up to $8000.00, while 10 subjects (22.2%) had received neither internal nor external funding. In 2009, 28.1% of entry-level faculty respondents reported receiving no grants [12], perhaps reflecting changing national economic factors more so than faculty productivity. The regression model derived from this data set (Figure 4, *p *= 0.007, m = $5870 per citation, y-intercept of $288 000, R^2^ = 0.159) predicted with 95% certainty a researcher with 200 citations has received $1 462 000 ±$910 666.13 in external funding. Determining the ratio of faculty citations to funding received may be a useful metric of faculty research efficiency. The promoting institution’s Carnegie Classification did not significantly correlate overall with the faculty research productivity metrics due to increases in the variance at larger institutions. Increasing statistical power, perhaps obtained through a nationwide or survey study, may overcome this shortfall.

While none of the metrics correlated significantly with the years before promotion, this may be because many universities and institutions are moving toward a strict tenure-track timeline. The benefit for institutions is that this approach provides a standard timeframe during which faculty compete. The pitfalls of this approach is that not all areas of research can result in the same metric outcomes while still being able to provide a strong impact within and even outside of their field. In addition, while none of the metrics significantly correlated, it should be noted that three interrelated metrics – total citations, number of publications, and h-index – trended lower for faculty promoted later, indicating that research-based achievement may be considered for early promotion but non-research related achievement such as teaching, administrative, and service activities may be considered for longer-standing faculty.

The process of creating standard metrics for evaluating faculty within and across disciplines has a long history with a range of different proposed approaches while attempting to control for a variety of factors including discipline-specific citation practices [13], length of the researcher’s career [3,14], the relative value of co-authorship, the database in which an article’s journal is indexed, number of self-citations, and chance authorship. In 2005, the h-index [3] was introduced and was quickly incorporated as a means to control for chance authorship. The h-index attempts to identify a researcher’s career impact while excluding outlying successes and failures; but the h-index is also criticized, especially if used as a sole metric for comparing researchers across disciplines due to variations between disciplines [15]. Other metrics exist, but any such indicator has its shortcomings and also influences a researcher’s decisions in publication of scientific data or how much risk to take in a research career.

We compared the number of citations of each article and the impact factor of the journal in which the article was published. These data indicate that the number of citations an article receives is neither closely nor significantly correlated to the five-year impact factor of the journal (Figure 6). This can be interpreted in multiple ways and is potentially due to a number of factors including access to certain journals, dissemination of research findings such as at conferences, and other factors that rely on the social nature of peer-reviewed scientific research. As the journal impact factor metric is designed to indicate the average number of citations per article published in a journal, the journal impact factor may be derived from non-normally distributed rates of citation. The ratio of an article’s total citations to the journal’s impact factor may be useful to identify the popularity of an article relative to other articles in that journal. It should be noted that the year in which an article was published correlated significantly with the number of citations it received (*p *= 2.1 × 10^−8^, m = 1.77, R^2^ = 0.079), as one would expect from the increased exposure that more time generally allows. One factor, not evaluated here, may be the collaborative network a researcher has cultivated within and outside the subject area which may broaden scope and enhance exposure for the researcher’s work. Institutional or geographical roadblocks to collaboration or dissemination may affect the impact of research in ways faculty are unable to influence.

One of the shortfalls of using publicly available vitae is that we cannot identify the specific factors considered by individual promotion and tenure committees which may play a greater role than quantitative metrics of research productivity. We are also unable to positively identify a cohort of faculty that did not successfully compete for promotion and tenure and thus are unable to determine if any of the measured factors were different for such an ‘unsuccessful’ cohort. Based on the broad range of measures observed for successfully promoted faculty, and the lack of significance of linear regressions with respect to the number of years at the assistant professor level until promotion was earned, these measures of research productivity seem insufficient to adequately explain the justifications for promotion. Research productivity as determined by these metrics is only one of a multitude of factors used by promotion and tenure committees. Teaching effectiveness and student perceptions are frequently considered as well, and these were not included in this analysis because the measures used by the institutions are not publicly available. Service projects and administrative functions may also be taken into account during the promotion and tenure process, and these were likewise unavailable for this study. According to the aggregate program data supplied by CAPTE, teaching makes up an average of 50.9% of the workload for core faculty in DPT programs, and scholarship only accounts for 21.1% [6]. Furthermore, while the data in this paper only reflects promotions in the southeastern USA, the extensive range observed by these metrics suggests broad promotion criteria are in use within institutions, and regional differences may not overcome this variability. However, future studies should focus on identifying possible regional differences in the metrics of promoted faculty. For these reasons, we find it reasonable to conclude that promotion and tenure decisions are based on a faculty member’s whole contribution and fit within a program and not based on the quantitative research metrics of an individual faculty member in competition nationwide or even in relation to broader regional averages.

The results of this study describe the average research productivity of DPT faculty who have successfully attained promotion in the southeastern region of the USA. This provides insight into the level of productivity that has previously been considered appropriate for promotion, which can then be used as comparative data when developing a standard for promotion and tenure decisions in the southeastern region. This data can also be used by tenure-track faculty planning to apply for promotion and/or tenure to gauge how their current research productivity compares to previously successful applicants in the region. No metric in this study resulted in a significant correlation with years spent earning promotion, possibly in part due to institutions adhering to strict tenure-track timelines and institutions considering faculty holistically in terms other than only research productivity and grant-earning history. Although this is the case, descriptive statistics for the subjects were determined. This study did not address a number of important factors, such as the public/private status of the institution, the program budget, or the Title VII protected characteristics of faculty; and was limited by availability of publically available CVs. Furthermore, the results of this study are specific to the southeastern USA, but regional data allows readers to compare findings in their current geographical location and to identify possible regional trends in relation to economic data.
